# MRI of the ‘Tiger’: a case series

**DOI:** 10.1093/ehjcr/ytaf461

**Published:** 2025-09-23

**Authors:** Johannes M I H Gho, Pieter T G Bot, Rick Halbmeijer, Cihal Gürlek, Alexander Hirsch

**Affiliations:** Department of Cardiology, Thorax Center, Cardiovascular Institute, Erasmus MC, Dr. Molewaterplein 40, Rotterdam 3015 GD, The Netherlands; Department of Radiology and Nuclear Medicine, Erasmus MC, Dr. Molewaterplein 40, Rotterdam 3015 GD, The Netherlands; Department of Cardiology, Spaarne Gasthuis, Boerhaavelaan 22, Haarlem 2035 RC, The Netherlands; Department of Cardiology, Beatrixziekenhuis, Banneweg 57, Gorinchem 4204 AA, The Netherlands; Department of Cardiology, Spaarne Gasthuis, Boerhaavelaan 22, Haarlem 2035 RC, The Netherlands; Department of Cardiology, Onze Lieve Vrouwe Gasthuis, Oosterpark 9, Amsterdam 1091 AC, The Netherlands; Department of Cardiology, Adrz, ‘s-Gravenpolderseweg 114, Goes 4462 RA, The Netherlands; Department of Cardiology, Thorax Center, Cardiovascular Institute, Erasmus MC, Dr. Molewaterplein 40, Rotterdam 3015 GD, The Netherlands; Department of Radiology and Nuclear Medicine, Erasmus MC, Dr. Molewaterplein 40, Rotterdam 3015 GD, The Netherlands

**Keywords:** Cardiomyopathy, Cardiovascular magnetic resonance, Cardiac MRI, Multi-modality imaging, Case series

## Abstract

**Background:**

Sawtooth cardiomyopathy (CMP) or ‘Tiger heart’ is a rare form of CMP of which only several cases have been described in literature. Hereby, we present two cases with this phenotype using cardiovascular magnetic resonance (CMR).

**Case summary:**

The first case was asymptomatic and referred for family screening. Cardiovascular magnetic resonance showed non-dilated, non-hypertrophic left and right ventricles with good systolic function. Morphologic abnormalities were seen with multiple band-shaped muscular bridges from the septal to the inferior wall fitting ‘sawtooth CMP’. The second case had a recent stroke at young age and was referred for analysis regarding a possible source of cardiac embolism. Cardiovascular magnetic resonance showed a dilated left ventricle with a basal inferoseptal aneurysm and severely impaired systolic function. Late enhancement images showed transmural fibrosis apical inferior.

**Discussion:**

These two cases of ‘sawtooth CMP’ illustrate a varying clinical picture from asymptomatic with normal ventricular function to dilated CMP with severely impaired systolic function. Reviewing current literature, we found several other case reports on sawtooth CMP. Sawtooth CMP was associated with various clinical sequelae including ventricular dysfunction, arrhythmias, and stroke. Non-invasive cardiovascular imaging was essential in their diagnostic work-up. The morphologic findings have been suggested to be different from established criteria to diagnose hypertrophic CMP or left ventricular non-compaction. The long-term prognostic implications are currently unknown.

Learning points

- Sawtooth CMP or ‘Tiger heart’ is an extremely rare form of CMP of which only several cases have been described.
- Sawtooth CMP was associated in several cases with various clinical sequelae including ventricular dysfunction, arrhythmias, and stroke. Non-invasive cardiovascular imaging was essential in their diagnostic work-up.
- The long-term prognostic implications are currently unknown. Future studies are needed to elucidate potential genetic causes and to investigate possible overlap between non-compaction or excessive trabeculation and sawtooth CMP.

## Introduction

Sawtooth cardiomyopathy (CMP) or ‘Tiger heart’ is a rare form of CMP of which only several cases have been described in literature since 2009.^1^ Morphologically, it is characterized by multiple band-shaped muscular bridges of compact myocardium attached to the septum and the ventricular wall resembling a sawtooth pattern or ‘Tiger stripes’. Hereby, we present two cases with this phenotype using cardiovascular magnetic resonance (CMR).

## Case presentation

### Case 1

**Figure 1:**
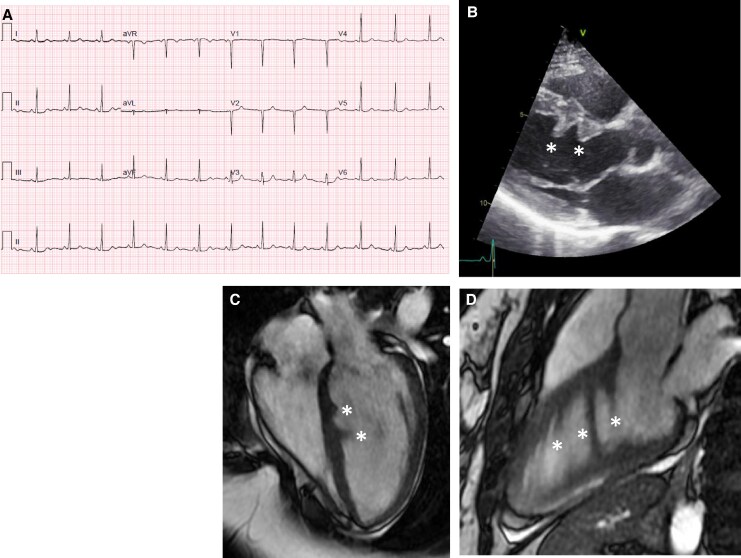
Case 1. Electrocardiogram (ECG) (*A*). Echocardiography parasternal long-axis view showing prominent muscular bundles (*) in a ‘sawtooth’ pattern (*B*). Cardiovascular magnetic resonance four-chamber view (*C*) and modified three-chamber view (*D*) showing multiple band-shaped muscular bridges from the septal to inferior wall (basal to apical) as seen in sawtooth cardiomyopathy.

A 32-year-old female was referred to the cardiology outpatient clinic because of a positive family history of premature coronary artery disease and sudden cardiac death. She had no relevant past medical history, was asymptomatic, and had a good exercise tolerance. Her father had passed away suddenly at age 63 because of a cardiac arrest (atherosclerosis found during autopsy). Her 36-year-old brother is known with mild left ventricular (LV) hypertrophy (maximum septal wall thickness 14 mm on CMR) and a previous anterior myocardial infarction. Her maternal grandfather suddenly died at age 65 (unknown cause). Clinical examination was unremarkable (normal heart sounds, S1, S2, no murmurs, blood pressure 135/86 mmHg) and did not show signs of heart failure. Her electrocardiogram (ECG) showed sinus rhythm with normal conduction times and absent R waves in V1–V2 (*Figure 1*).

Echocardiography showed a good systolic LV function with prominent muscular bundles in the left ventricle (see Supplementary material online, *Movies S1* and *S2*) and no valvular disease. Additional Holter recording showed no ventricular arrhythmias or conduction disturbances.

Cardiovascular magnetic resonance showed non-dilated, non-hypertrophic left and right ventricles with good systolic function. Morphologic abnormalities were seen with multiple band-shaped muscular bridges from the septal to the inferior wall (basal to apical) fitting sawtooth CMP (*Figure 1*). There was mid-wall late gadolinium enhancement (LGE) in the basal septum, with no signs of previous myocardial infarction. Cine movies are presented in the Supplementary material.

She was referred for genetic testing with follow-up in our outpatient clinic. Using whole exome sequencing, we did not find a genetic aetiology. A 3-year follow-up period until now has been unremarkable.

### Case 2

A 43-year-old male who suffered from a stroke was referred to the cardiologist for analysis regarding a possible source of cardiac embolism. He had a blank past medical history. His family history was negative for cardiovascular disease. The ECG showed sinus rhythm with a right axis deviation and a mild intraventricular conduction delay (QRS duration 114 ms), and his blood pressure was 129/88 mmHg. During Holter monitoring, a non-sustained ventricular tachycardia of 17 complexes was registered (max. 175 b.p.m.).

**Figure 2:**
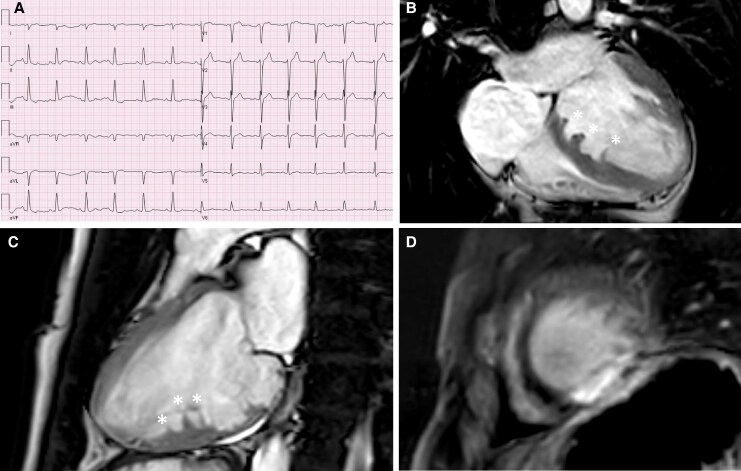
Case 2. Electrocardiogram (ECG) (*A*). Cardiovascular magnetic resonance four-chamber view (*B*) and modified two-chamber view (*C*) showing multiple band-shaped muscular bridges (*) from the anteroseptal to inferior wall (basal to mid-ventricular) fitting sawtooth cardiomyopathy. Late gadolinium enhancement image (*D*) showing signs of transmural fibrosis apical inferior.

Transthoracic echocardiographic imaging was severely hampered in quality because of the slender physique of the patient, and therefore, a CMR was performed. Cardiovascular magnetic resonance showed a dilated left ventricle with a basal inferoseptal aneurysm and severely impaired systolic function (end-diastolic volume/body surface area 158 mL/m^2^, ejection fraction 28%) (*Figure 2*). Morphologic abnormalities were seen with multiple band-shaped muscular bridges from the anteroseptal to inferior wall (basal to mid-ventricular) fitting sawtooth CMP. Late enhancement images showed transmural fibrosis apical inferior, without signs of LV thrombus. Cine movies are presented in the Supplementary material.

Because of the severely impaired systolic LV function and a non-specific LGE pattern, additionally, a FDG-PET-CT scan was performed to rule out cardiac sarcoidosis, but this showed no cardiac FDG uptake. Coronary angiography revealed normal coronary arteries, and the differential diagnosis included a thrombo-embolic infarction. Because of a high suspicion regarding a cardiac embolism together with the severely impaired LV systolic function, oral anticoagulation was initiated besides an ACE-inhibitor, beta-blocker, and statin. During follow-up, a subcutaneous implantable cardioverter-defibrillator (S-ICD) was implanted. Genetic testing (CMP panel 56 genes) yielded no pathogenic mutations around 1 year after presentation.

## Discussion

These two cases of ‘sawtooth CMP’ with CMR illustrate a varying clinical picture from asymptomatic with normal ventricular function to dilated CMP with severely impaired systolic function. Reviewing current literature, we found several other case reports on sawtooth CMP (*n* = 15, including our two cases). The characteristics of these patients are presented in *Table 1* (references provided in the Supplementary material).

**Table 1:** Patient characteristics of all sawtooth cardiomyopathy cases described in the literature including the two patients presented in this report

Total cases	*n* = 15
Age at presentation (years) (mean ± SD)	35 ± 22
Female sex	4 (27%)
Non-sustained ventricular tachycardia	2 (13%)
ICD implanted	2 (13%)
Intraventricular conduction delay on ECG	6 (40%)
Transient ischemic attack (TIA)/stroke	3 (20%)
Late gadolinium enhancement on CMR	4 (27%)
Left ventricular ejection fraction (mean), reduced	56%, 8/15

The mean age of all patients was 35 years and 27% were female. Sawtooth CMP was associated with various clinical sequelae including ventricular dysfunction, arrhythmias, and stroke. Non-invasive cardiovascular imaging was essential in their diagnostic work-up. The morphologic findings have been suggested to be different from established criteria to diagnose hypertrophic CMP or LV non-compaction. Hypertrophic CMP is commonly characterized by LV asymmetric hypertrophy, and the associated apical-basal muscular bundles differ in orientation. Left ventricular non-compaction differs because it is characterized by a non-compact sponge-like, trabecular layer with prominent trabeculae. The long-term prognostic implications of sawtooth CMP are currently unknown. In case of systolic impairment, it is recommended to treat according to clinical guidelines. In our first case, who was asymptomatic with preserved systolic LV function, the sawtooth myocardium could be a benign morphological variant. In our second case, one could hypothesize that the sawtooth CMP could have played a causal role in stroke due to LV dysfunction, resulting in cardiac thrombus formation and embolism. The first case report with sawtooth CMP which reported on genetic testing did not identify a pathogenic mutation.^2^ More recently, a report that investigated several families with missense variants in *FLNC* showed a high prevalence of sawtooth appearance of the myocardium.^3^ Future studies are needed to elucidate potential underlying pathophysiologic and genetic causes and to investigate possible overlap between non-compaction or excessive trabeculation and sawtooth CMP.

## Lead author biography


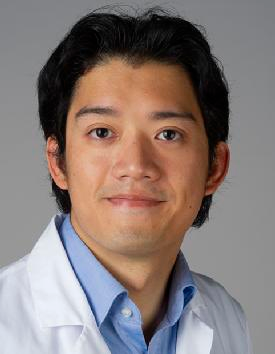


Johannes Gho was born in the year of the ‘Tiger’ and trained in cardiology at the University Medical Center Utrecht, Utrecht, the Netherlands. His PhD degree entitled ‘Opportunities in the Failing Heart’ encompassed epidemiologic studies on heart failure, studies aimed to improve cardiac fibrosis detection by optimizing CMR and histological analyses, identifying (epi)genetic pathways leading to heart failure and translational therapeutic studies. He has been involved as an author in multiple publications regarding cardiovascular imaging and (genetic) cardiomyopathies. He recently completed a fellowship non-invasive cardiovascular imaging at the Erasmus MC, Rotterdam, the Netherlands. He is currently working as an imaging cardiologist in the Netherlands.

## Supplementary Material

ytaf461_Supplementary_Data

## Data Availability

Data will be made available on request.

## Notes

**Consent:** The authors confirm that written consent for submission and publication of this case report including images and associated text has been obtained from the patients. This publication complies with the COPE guidelines on ethical publication.

**Funding:** None declared.
