# Supplementary material for: MRI of the ‘Tiger’: a case series
Source: Eur Heart J Case Rep. 2025 Sep 23;9(11):ytaf461. doi: 10.1093/ehjcr/ytaf461 (PMC12582066; doi:10.1093/ehjcr/ytaf461)
Supplement: ytaf461_Supplementary_Data [file ytaf461_supplementary_data.zip › Tiger_draft_supplemental_references.docx]

**Supplemental Material**

Supplemental references^1-12^

1. Davlouros PA, Danias PG, Karatza AA, Kiaffas MG, Alexopoulos D. Saw-tooth cardiomyopathy. *J Cardiovasc Magn Reson*. 2009;11:54.

2. Rafiq I, Ghosh-Ray S, Curtin J, Williams I. Images in cardiology. A previously undescribed variant of isolated left ventricular noncompaction. *J Am Coll Cardiol*. 2010;56:741.

3. Parameswaran AC, Cheong BY. Tiger heart: a variant of isolated left ventricular noncompaction? *Tex Heart Inst J*. 2012;39:444-445.

4. Aneja A, Hoffman T, Roble S, Raman S. Sawtooth cardiomyopathy. 2013. <https://scmr.org/page/COW1311>

5. Cardoso BP, Trigo C, Jalles Tavares N, Pinto FF. Sawtooth cardiomyopathy: A rare cause of heart failure. *Rev Port Cardiol*. 2017;36:875-876.

6. Bailly MT, Vasile T, Berthelot E, Abdo A, El Hatimi S, Jourdain P, et al. [Saw tooth cardiomyopathy: How to better diagnose?]. *Ann Cardiol Angeiol (Paris)*. 2020.

7. Chenaghlou M, Kasaei M, Taghavi S, Amin A, Naderi N. Saw tooth cardiomyopathy: a case report. *ESC Heart Fail*. 2020;7:325-328.

8. Halioui M, Grigoratos C, Todiere G, Aquaro GD, Barison A. Mitral valve prolapse and partial saw-tooth cardiomyopathy: an unusual combination. *J Cardiovasc Med (Hagerstown)*. 2020;21:829-830.

9. Proukhnitzky J, Garot J, Bordet C, Legrand L, Ader F, Richard P, et al. Saw-Tooth Cardiomyopathy: Clinical Presentation and Genetic Analysis. *JACC Case Rep*. 2020;2:1205-1209.

10. Wegner FK, Diller GP, Eckardt L, Reinecke H, Orwat S. A case of 'tiger heart': a distinct variant of left ventricular non-compaction. *Eur Heart J Cardiovasc Imaging*. 2020;21:1434.

11. Garcia-Ropero A, Antonakaki D, Savvatis K. Saw-tooth cardiomyopathy: cardiomyopathies baring their teeth. *Rev Esp Cardiol (Engl Ed)*. 2021.

12. Berarducci J, Armenta-Moreno JI, Espinola-Zavaleta N, Cano-Zarate R, Gutierrez-Solana-Ossa AV, Keirns C. Computed tomography assessment of saw-tooth cardiomyopathy: a case series. *Eur Heart J Case Rep*. 2022;6:ytab528.

Systematic literature search updated on 10th March 2025

PubMed search

sawtooth[Title/Abstract] OR saw-tooth[Title/Abstract] OR Tiger[Title/Abstract]

AND

cardiomyopathy[Title/Abstract] OR CMP [Title/Abstract] OR heart[Title/Abstract]

Exclusion criteria: review article, editorial, animal study

155 results

*Screening Title and Abstract*

11 relevant full-text articles
