# Supplementary material for: MRI of the ‘Tiger’: a case series
Source: Eur Heart J Case Rep. 2025 Sep 23;9(11):ytaf461. doi: 10.1093/ehjcr/ytaf461 (PMC12582066; doi:10.1093/ehjcr/ytaf461)
Supplement: ytaf461_Supplementary_Data [file ytaf461_supplementary_data.zip › Tiger_draft_supplemental_movies_1_v2.pptx]

## Slide 1
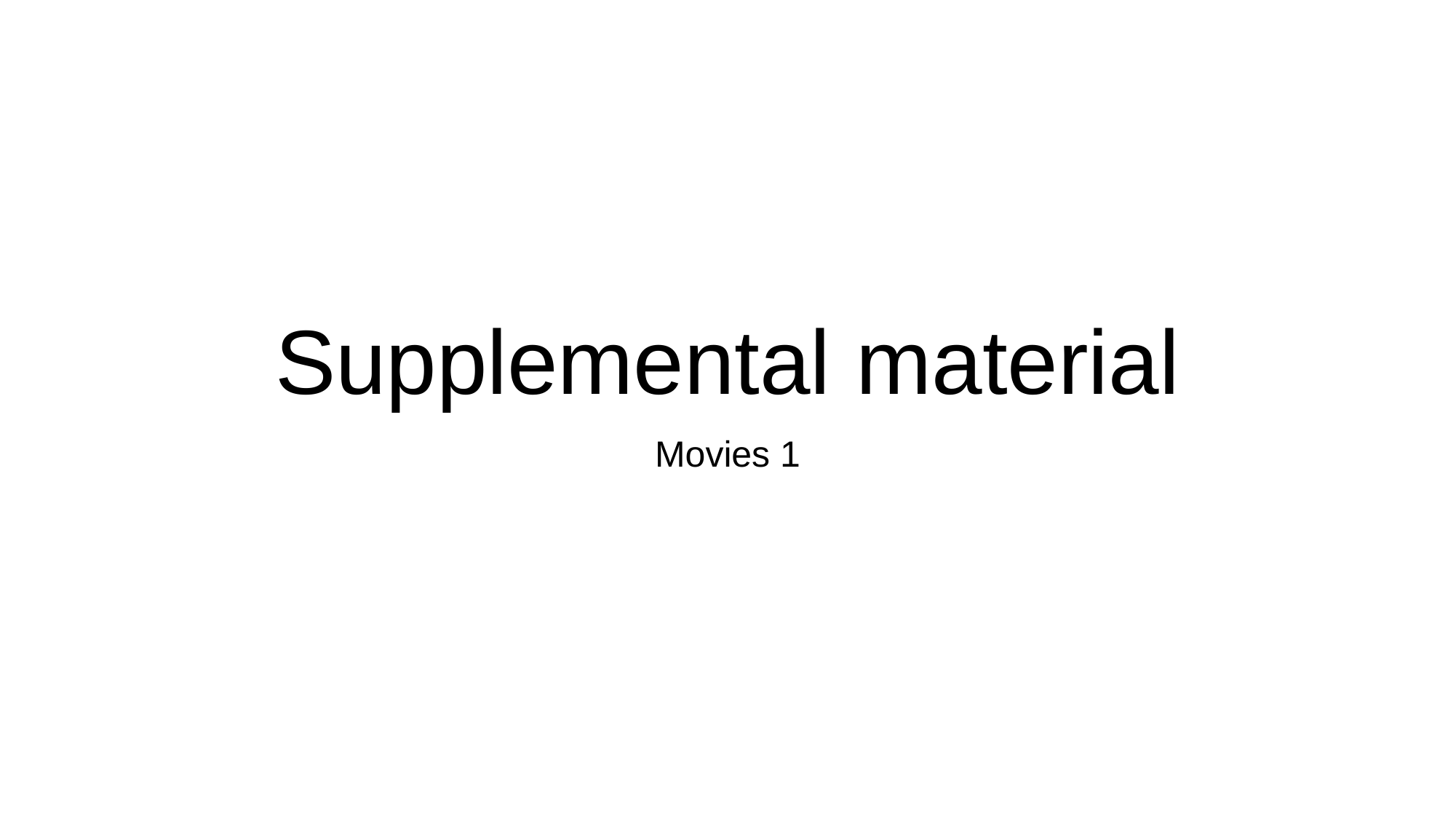

# Supplemental material
Movies 1

## Slide 2
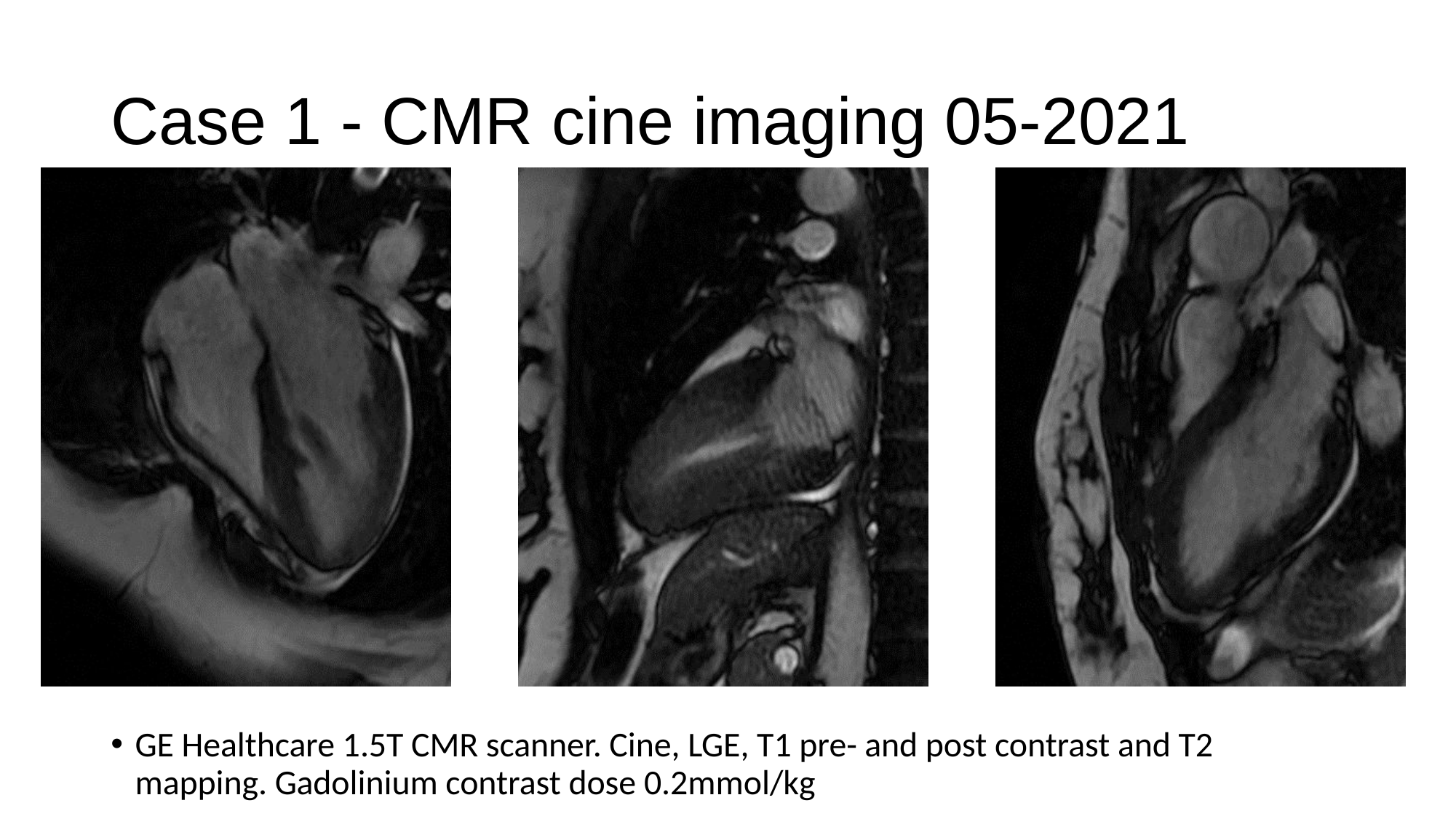

# Case 1 - CMR cine imaging 05-2021
GE Healthcare 1.5T CMR scanner. Cine, LGE, T1 pre- and post contrast and T2 mapping. Gadolinium contrast dose 0.2mmol/kg

## Slide 3
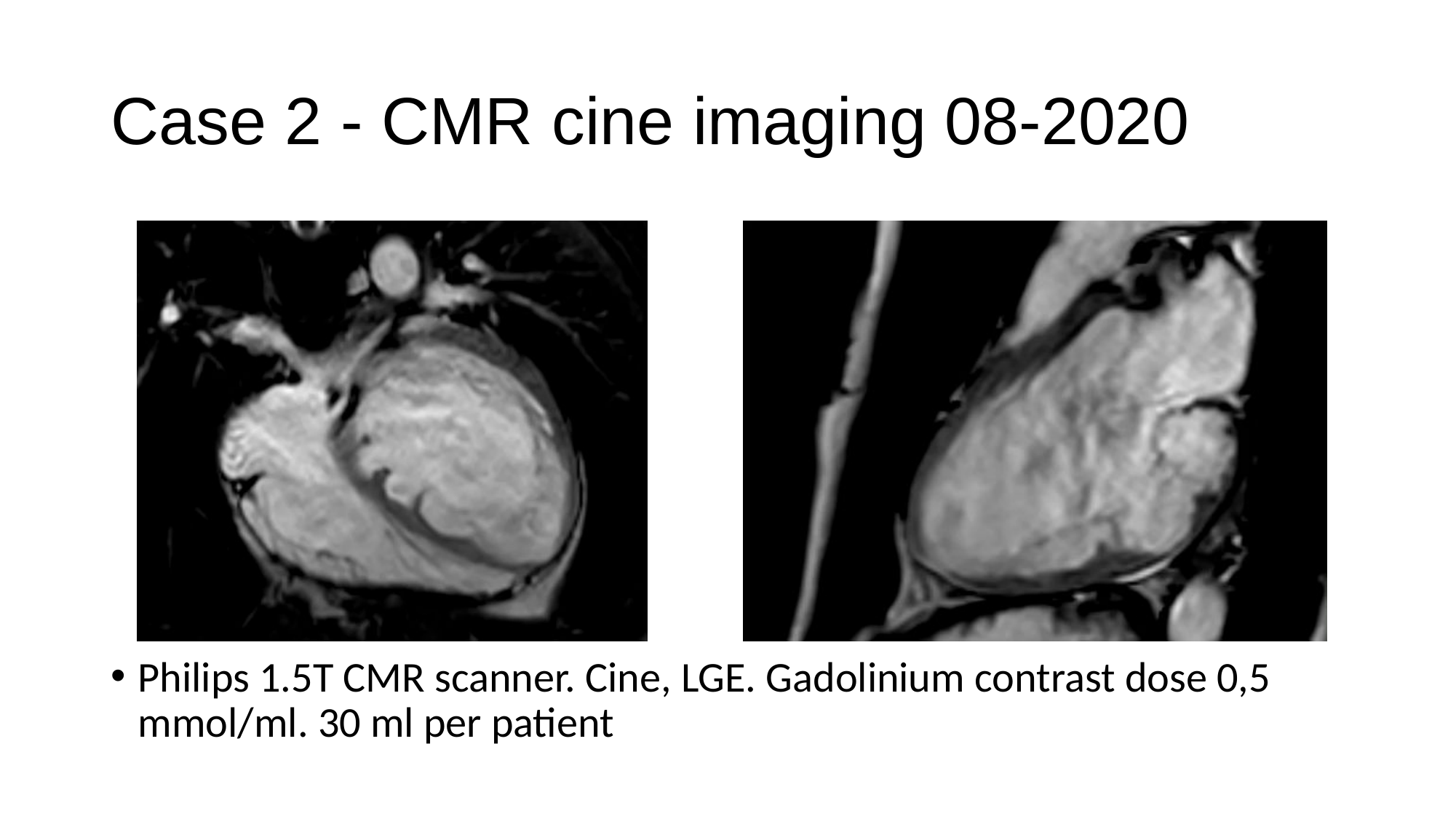

# Case 2 - CMR cine imaging 08-2020
Philips 1.5T CMR scanner. Cine, LGE. Gadolinium contrast dose 0,5 mmol/ml. 30 ml per patient
