# Supplementary material for: MRI of the ‘Tiger’: a case series
Source: Eur Heart J Case Rep. 2025 Sep 23;9(11):ytaf461. doi: 10.1093/ehjcr/ytaf461 (PMC12582066; doi:10.1093/ehjcr/ytaf461)
Supplement: ytaf461_Supplementary_Data [file ytaf461_supplementary_data.zip › Tiger_draft_supplemental_movies_2_v2.pptx]

## Slide 1
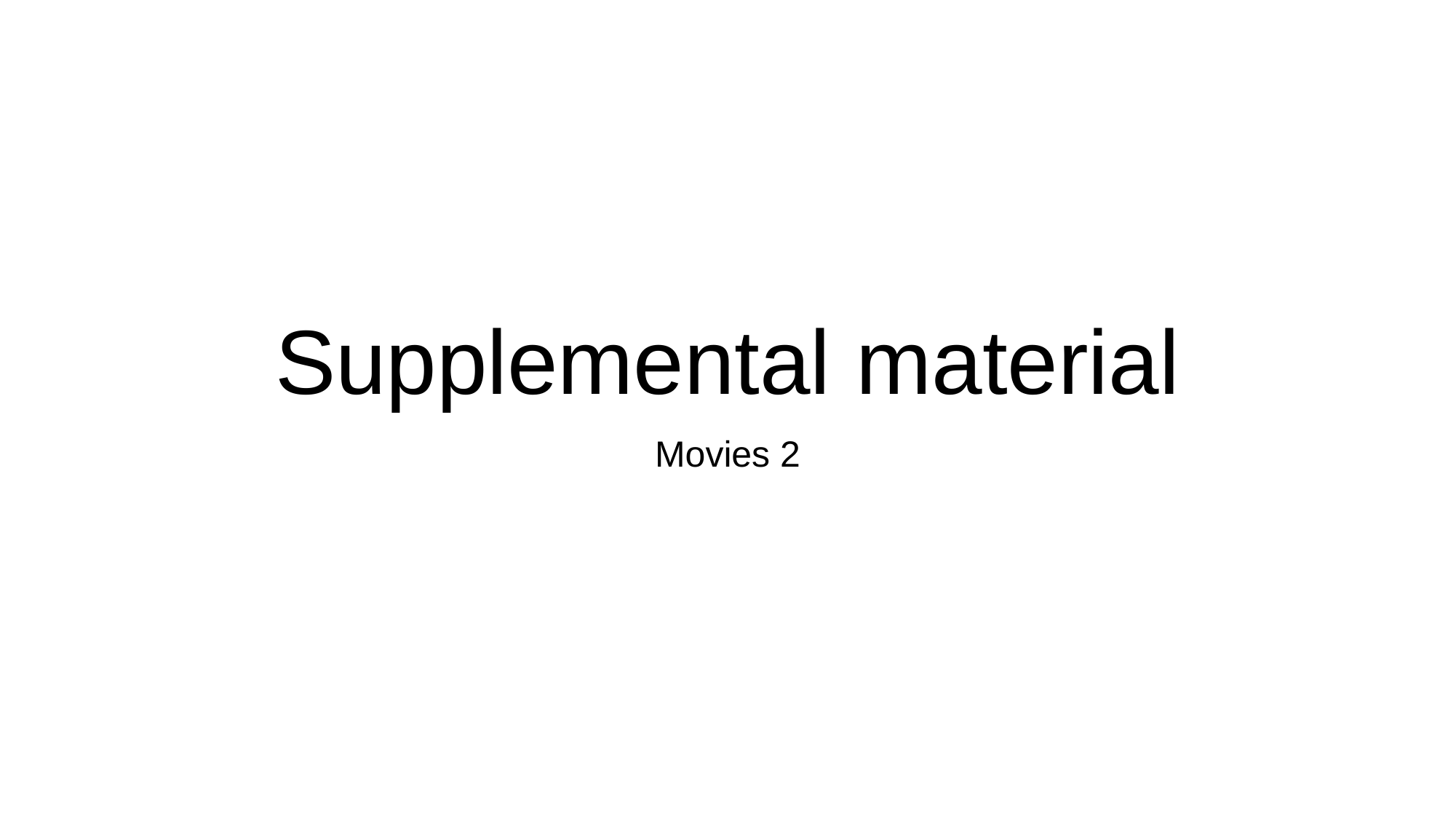

# Supplemental material
Movies 2

## Slide 2
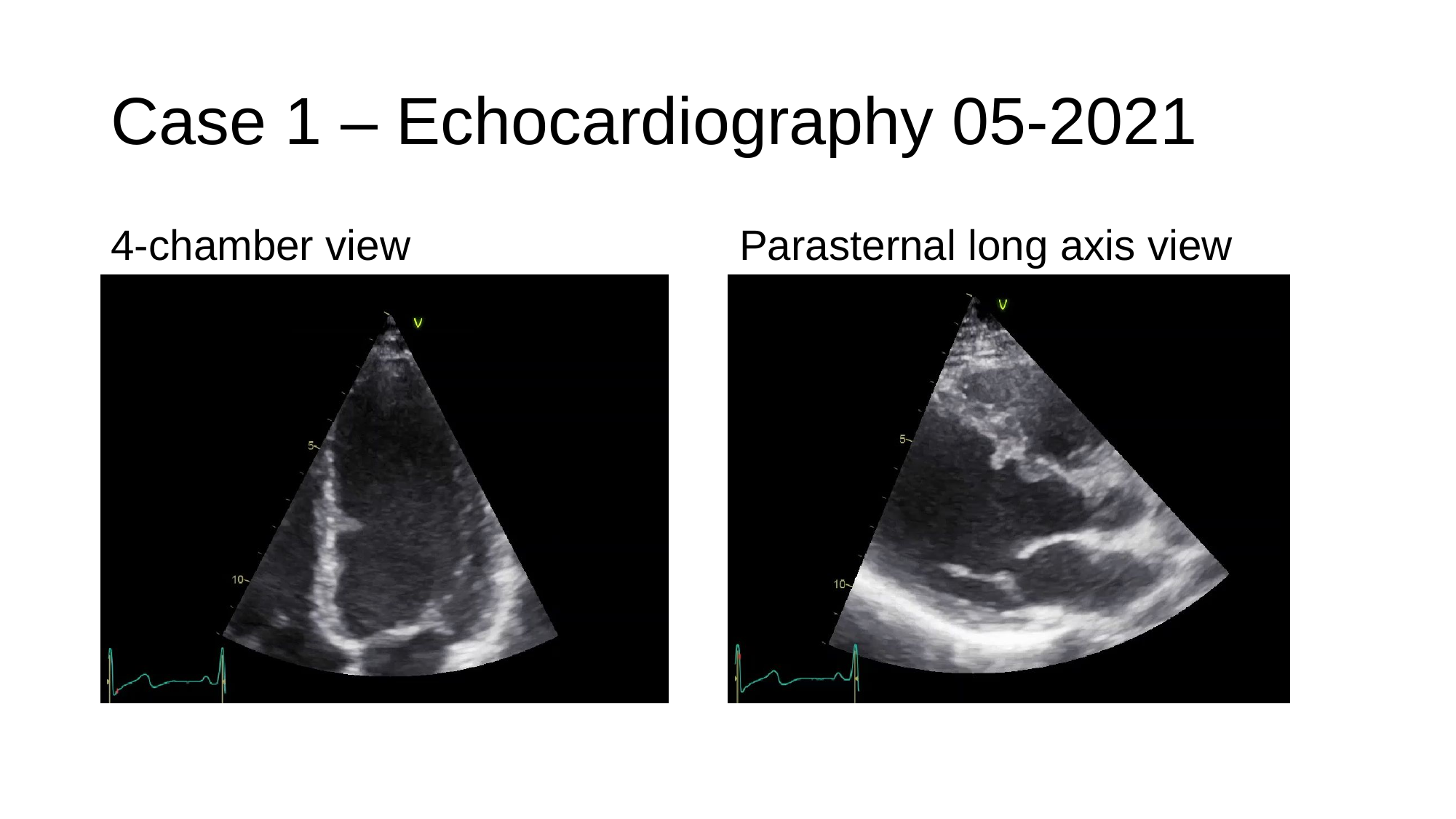

# Case 1 – Echocardiography 05-2021
4-chamber view			 Parasternal long axis view

## Slide 3
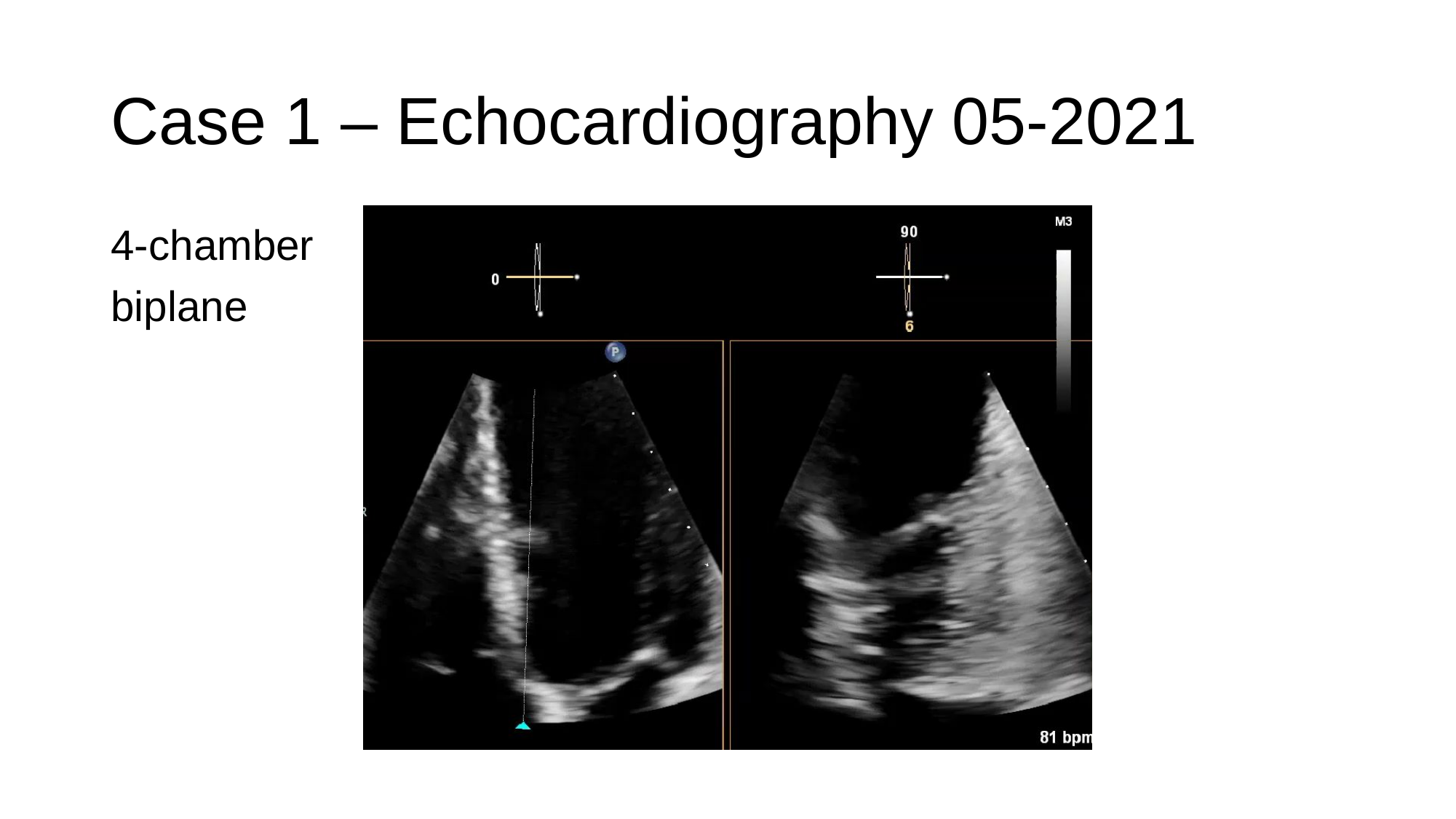

# Case 1 – Echocardiography 05-2021
4-chamber
biplane
